# Matrix Metalloproteinase-9-Dependent Release of IL-1*β* by Human Eosinophils

**DOI:** 10.1155/2019/7479107

**Published:** 2019-02-17

**Authors:** Stephane Esnault, Elizabeth A. Kelly, Sean H. Johnson, Larissa P. DeLain, Madeline J. Haedt, Andrea L. Noll, Nathan Sandbo, Nizar N. Jarjour

**Affiliations:** University of Wisconsin-Madison School of Medicine and Public Health, Department of Medicine, Division of Allergy, Pulmonary and Critical Care Medicine, Madison, WI, USA

## Abstract

Asthma is often associated with airway eosinophilia, and therapies targeting eosinophils are now available to treat severe eosinophilic asthma. Eosinophilic asthma is often due to a type-2 immune response and production of IL-5, which leads to eosinophilopiesis and recruitment of mature eosinophils in the airways. A concomitant type-2 and type-17 response has been reported in some individuals. IL-17 may be enhanced by IL-1*β* production and can lead to neutrophilic inflammation. In fact, both eosinophilic and neutrophilic (mixed granulocytic) inflammation are simultaneously present in a large population of patients with asthma. In monocyte/macrophage cell populations, release of mature IL-1*β* occurs via toll-like receptor ligand-induced activation of the inflammasome. Within the inflammasome, a cascade of events leads to the activation of caspase-1, which cleaves pro-IL-1*β* protein into a mature, releasable, and active form. We have demonstrated that eosinophils can release IL-1*β* in a Toll-like receptor ligand-independent fashion. The objective of this study was to determine the mechanisms underlying the production and maturation of IL-1*β* in cytokine-activated eosinophils. Using eosinophils from circulating blood and from bronchoalveolar lavage fluid after an airway allergen challenge, the present study demonstrates that cytokine-activated eosinophils express and release a bioactive form of IL-1*β* with an apparent size less than the typical 17 kDa mature form produced by macrophages. Using a zymography approach and pharmacological inhibitors, we identified matrix metalloproteinase-9 (MMP-9) as a protease that cleaves pro-IL-1*β* into a ~15 kDa form and allows the release of IL-1*β* from cytokine-activated eosinophils. Therefore, we conclude that activated eosinophils produce MMP-9, which causes the release of IL-1*β* in an inflammasome/caspase-1-independent manner. The production of IL-1*β* by eosinophils may be a link between the eosinophilic/type-2 immune response and the neutrophilic/type-17 immune response that is often associated with a more severe and treatment-refractory type of asthma.

## 1. Introduction

Eosinophils are leukocytes present and active in tissues during a variety of disease manifestations, including allergy and asthma. Eosinophils can release toxic proteins and inflammatory mediators (cytokines, chemokines, and lipids) [1], and their presence in the airway is often associated with more severe asthma [2, 3]. Typically, eosinophilic asthma is linked with a type-2 immune response characterized by the production of IL-4, IL-5, and IL-13. IL-5 and IL-13 are both generated by innate lymphoid cells (ILC) and lymphocytes in response to danger signals and allergens [4]. Distinctively, neutrophilic asthma is associated with the inflammasome/IL-1 pathway and a type-17 immune response [5, 6] that contributes to a treatment-refractory asthma phenotype [7]. However, the dichotomy between eosinophilic versus neutrophilic asthma is not absolute since mixed granulocytic asthma is observed in ~20% of the severe asthmatic population [8, 9]. Moreover, CD4+ T lymphocytes producing both type-2 and type-17 cytokines have been reported in the blood and airways of asthmatic patients [10, 11]. Notably, Seys et al. have described the coexpression of type-2 and type-17 cytokines in the airways of subjects with poorly controlled asthma [12]. Interestingly, these type-2/type-17 “high” patients also displayed higher concentrations of IL-1*β* in bronchoalveolar lavage (BAL) fluid that was highly correlated with the numbers of airway Th2/Th17 cells [13]. Leaker et al. reported that a nasal allergen challenge induced both type-2 inflammation and the production of IL-1*β* [14]. In addition, we recently showed that although the sputum expression level of IL-1/IL-17 molecular markers most strongly correlated with neutrophilia, all type-2 and type-17 markers, as well as the IL-1 receptor expression levels tended to correlate with each other, indicating a lack of clear-cut separation between these different types of immune responses in asthma [6].

The IL-1 receptor is present on Th17 lymphocytes [15], and IL-1*β* alone can induce the expression of the master Th17 differentiation factor RAR related orphan receptor C (RORC) in naïve CD4+ T [16]. IL-1*β* also increases IL-17 production by memory T lymphocytes [17, 18] and activates ILC type-2 (ILC2) [19]. The importance of IL-1*β* in asthma is highlighted by the observations that IL-1*β* is elevated in BAL fluid and sputum [20, 21]; it is associated with nocturnal asthma [22]; and the expression of its receptor (IL-1R1) is positively correlated to stress markers in asthmatic patients [23]. The expression of the IL-1 receptor on fibroblasts and epithelial and airway smooth muscle cells [24–26] suggests that IL-1 may play a role in lung tissue remodeling and loss of pulmonary function in asthma [27]. Thus, the IL-1 pathway has been proposed as a potential therapeutic target in asthma [28].

Macrophages are a principle source of inflammasome-dependent IL-1*β* generation [29, 30]. In macrophages, IL-1*β* is produced as a 31 kDa proform that is cleaved into a biologically active 17 kDa mature form. These processes are dependent on cell activation by both a toll-like receptor and adenosine triphosphate (ATP), which activate the inflammasome [31]. An inflammasome is composed of a nucleotide-binding oligomerisation domain (NOD), leucine rich repeat and pyrin domain containing (NLRP), which recruits and activates caspase-1, which in turn cleaves the pro-IL-1*β* into an active and releasable form [32].

Eosinophils isolated from the gastrointestinal tract of mice produce large amounts of IL-1*β* [33]. IL-1*β* is also released by mouse eosinophils from fibrotic liver tissues in a caspase-1-mediated pyroptosis manner [34]. In humans, blood eosinophils activated with monosodium urate and toll-like receptor ligands release IL-1*β* [35, 36]. We have previously shown that human eosinophils cultured *in vitro* with a low (prosurvival) amount of GM-CSF could spontaneously release enough IL-1*β* to increase the production of IL-17 by CD4+ T lymphocytes [18]. However, the mechanisms of IL-1*β* production and maturation in eosinophils remain unknown. In the present study, we have used an established model of potent eosinophil activation by concurrent treatment with IL-3 and TNF-*α* [37, 38] to induce the production of IL-1*β* and thereby examine the mechanisms mediating its expression, maturation, and release.

## 2. Materials and Methods

### 2.1. Human Subjects

Twenty-eight blood donors participated in the study. All had a history of allergy (at least one positive skin prick test) with or without rhinitis or mild asthma. Subjects with prescriptions for low doses of inhaled corticosteroids did not use their corticosteroids the day of the blood draw. Twenty-eight subjects participated in this study. The University of Wisconsin-Madison Health Sciences Human Subjects Committee approved the study protocols and informed written consent was obtained from each subject prior to participation.

### 2.2. Eosinophil Purification

Eosinophils were purified by negative selection as previously described [39]. Briefly, heparinized blood was diluted 1 : 1 in HBSS and was overlaid above Percoll (1.090 g/ml). After centrifugation at 700 × *g* for 20 min at room temperature, the mononuclear cells were removed from the plasma/Percoll interface and erythrocytes were eliminated from the cell pellet by hypotonic lysis with water. The remaining pellet was resuspended in 2% NCS in HBSS. Cells were then incubated with anti-CD16, anti-CD3, anti-CD14, and anti-Glycophorin-A beads from Miltenyi Biotec (San Diego, CA) and run through an AutoMACS (Miltenyi Biotec). Eosinophil preparations with purity > 99% and viability~98% were cultured the same day, ~6-8 h after the blood draw.

### 2.3. Bronchoalveolar Lavage (BAL) Fluid and Airway Eosinophil (BAL Eosinophil) Preparation

As previously described [40], BAL were performed 48 h after segmental bronchoprovocation with an allergen (SBP-Ag) in allergic subjects with mild asthma. BAL EOS were purified from BAL cells using a two-step Percoll gradient. EOS were collected from the 1.085/1.100 g/ml interface.

### 2.4. Eosinophil Culture

Eosinophils were cultured at 1-2 × 10^6^ cells/ml in RPMI-1640 containing HEPES, L-glutamine, 10% FBS, 1% antibiotic-antimycotic (Thermo Fisher Scientific, Waltham, MA, USA), and 10 *μ*g/ml ciprofloxacin hydrochloride (Bioworld/Thermo Fisher Scientific). Cells were stimulated with 10 ng/ml of a *β*c chain-signaling cytokine (IL-3 or IL-5) alone or in combination with 10 ng/ml of TNF-*α* for up to 72 h or as indicated. In specified experiments, eosinophils were preincubated (30 min) with pharmacological inhibitors of caspase-1 (Z-WEHD-FMK, Enzo Life Sciences Inc., Farmingdale, NY, USA) or MMP-9 (MMP-9 inhibitor 1, Enzo Life Sciences Inc.), both used at 5 *μ*M, or vehicle alone.

### 2.5. Quantitative Reverse Transcription Polymerase Chain Reaction (RT-qPCR)

As previously described [41], total RNA was extracted from eosinophils using the RNeasy Mini Kit (Qiagen, Valencia, CA, USA). The reverse transcription reaction was performed using the Superscript III system (Invitrogen/Life Technologies, Grand Island, NY, USA). mRNA expression was determined by qPCR using SYBR Green Master Mix (SABiosciences, Frederick, MD, USA) and human IL-1*β*- (forward: tggaccccttggtaaaagaca, reverse: gaagaaatcagtagagctatgaaacaaataag) specific primers. Primers were designed using Primer Express Software v3.0 (Applied Biosystems, Carlsbad, CA, USA) and blasted against the human genome to determine specificity using http://www.ncbi.nlm.nih.gov/tools/primer-blast. The reference gene, *β*-glucuronidase ((GUSB), forward: caggacctgcgcacaagag, reverse: tcgcacagctggggtaag), was used to normalize the samples. Standard curves were performed and efficiencies were determined for each set of primers. Efficiencies ranged between 91 and 96%. Data are expressed as fold change using the comparative cycle threshold (∆∆Ct) method and the values presented are fold change = (2^−*∆∆*Ct^).

### 2.6. IL-1*β* mRNA Decay

As previously described [38], the transcription inhibitor 5,6-dichloro-1-*β*-D-ribofuranosylbenzimidazole (DRB; 25 *μ*g ml/ml) was added to eosinophil cultures 4.5 h after the addition of TNF-*α* plus IL-3 (cytokines at 10 ng ml/ml), and eosinophils were harvested 30 and 90 min thereafter. *IL1B* mRNA levels present immediately before the addition of DRB (*T* = 0 h) were set to 100%. The percentage of *IL1B* mRNA remaining compared with *T* = 0 h was presented for each time point after the addition of DRB. The half-life of mRNA was defined as the time required to attain a 50% reduction of mRNA after DRB addition.

### 2.7. IL-1*β* Elisa

IL-1*β* concentration in cell-culture supernatants was determined using the R&D Systems DuoSet Development kit (DY201, Minneapolis, MN, USA), which detects mature IL-1*β* (<7% cross-reactivity with pro-IL-1*β*).

### 2.8. IL-1*β* Bioassay

A HEK-Blue™ IL-1*β* reporter cell line (InvivoGen, San Diego, CA, USA) that expresses a NF-*κ*B/AP-1-inducible SEAP reporter gene was used to measure IL-1*β* bioactivity in eosinophil-conditioned media. The reporter cells were incubated overnight with either conditioned media from resting or TNF-*α* plus IL-3-activated eosinophils, or a recombinant human (rh) mature IL-1*β* (#201-LB R&D Systems, Minneapolis, MN, USA) used at different concentrations. The IL-1*β*-induced NF-*κ*B signaling pathway was monitored using spectrophotometer absorbance values at 650 nm. Bioactivity in eosinophil cultures was interpolated using the standard curve that was created using absorbance values at 605 nm versus known concentrations of recombinant human IL-1*β*. To test the specificity of IL-1*β*, a soluble recombinant human IL-1*β* receptor antagonist, rhIL1RA (0.4 *μ*g/ml; R&D Systems), was added on HEK cells 30 min before the conditioned media.

### 2.9. Western Blot

Eosinophil lysates were prepared by adding 1-2 million eosinophils to 35 *μ*l of RIPA lysis buffer (Cell Signaling Technology, Danvers, MA, USA) containing 20 mM Tris HCl (pH 7.5), 150 mM NaCl, 1 mM EDTA, 1 mM EGTA, 2.5 mM sodium pyrophosphate, 1 mM sodium orthovanadate, 1 *μ*g/ml leupeptin, 1 mM *β*-glycerophosphate, 1% sodium deoxycholate, 1% NP-40, and 0.1% SDS. PMSF (1 mM) and a cocktail of mammalian protease inhibitors including AEBSF, aprotinin, bestatin, E-64, leupeptin, and pepstatin A (P8340, Sigma-Aldrich Corp., St. Louis, MO, USA) were added just prior to use. Eosinophil culture supernatants were concentrated 12.5-fold using Amicon Ultra centrifugal filters of 50,000 and 3,000 kDa (Millipore/Sigma-Aldrich Corp., Burlington, MA, USA). Lysates and concentrated culture supernatants were resolved by electrophoresis on 15% SDS-polyacrylamide gels and transferred to a PVDF membrane. Protein was detected with a polyclonal goat antibody to an epitope mapping at the C-terminus of IL-1*β* (C-20, Santa Cruz Biotechnology, Dallas, Texas, USA) or a polyclonal rabbit antibody to the 17 kDa mature form of human IL-1*β* (Cell Signaling Technology, Danvers, MA, USA), followed by the use of a secondary HRP-conjugated donkey anti-goat antibody (Santa Cruz Biotechnology) or secondary HRP-conjugated anti-rabbit IgG antibody (Pierce/Thermo Fisher Scientific, Rockford, IL, USA). *β*-Actin was used as a loading control. Mouse monoclonal anti-*β*-actin was purchased from Sigma-Aldrich Corp. Imaging was performed with a SuperSignal™ West Femto chemiluminescent substrate (Life Technologies, Grand Island, NY, USA) on an ImageQuant™ LAS 4000 imager (GE Healthcare, Piscataway, NJ, USA). For western blots using BAL fluids, two rhIL-1*β* were mixed to visualize the pro- and mature form of IL-1*β*. The rhIL-1*β* proform (~17 kDa) was from Sino Biological, Wayne, PA, USA, and the mature form (~31 kDa) was from HumanZyme Inc., Chicago, IL, USA.

### 2.10. Zymography

For cell lysate preparation, two million inactivated or TNF-*α* plus IL-3-activated eosinophils were added to 35 *μ*l of RIPA lysis buffer (Cell Signaling Technology) supplemented with 0.1% SDS and 1% Triton X-100. PMSF (1 mM) and the protease inhibitors aprotinin (10 *μ*g/ml), leupeptin (2 *μ*g/ml), and pepstatin (20 *μ*g/ml) were added just prior to use. The cell suspension was sonicated 2 times with 2 second pulses (output setting 2, Sonicator 3000, Misonix, Farmingdale, NY, USA), repeatedly passed through a syringe (28-gauge needle), and clarified by centrifugation (12,000 × *g*/10 min/4°C). Zymography was performed as previously described [37, 42] with the following modifications. One *μ*g/ml recombinant proIL-1*β* (Sino Biological/InvivoGen, San Diego, CA USA) was copolymerized into a 12% polyacrylamide gel for 48 h. Cell lysates were electrophoresed under nonreducing conditions on the pro-IL-1*β*-containing gel. The pro-IL-1*β* zymograms were renatured and incubated overnight under conditions compatible with the activation of caspases (50 mM Tris pH 7.2, 200 mM NaCl, 0.02% Brij, and 0.5 EDTA) or MMPs (50 mM Tris, 200 mM NaCl, 5 mM CaCl, 1 *μ*M ZnCl, and 0.005% Brij).

### 2.11. Detection of Proteases for Pro-IL-1*β* by ELISA and Mass Spectrometry-Based Proteomics

Each lane of the pro-IL-1*β* zymogram gel was excised and sequentially cut into 1 mm slices. The size of proteins in each gel slice was calculated by determining and plotting the relative migration distance (Rf = migration distance of the protein/migration distance of the dye front) of each molecular weight marker against its molecular mass and interpolating the apparent size based on the Rf of the center of the gel slice. Proteins were allowed to diffuse out of the gel by incubating the slices overnight at 4°C with 55 *μ*l PBS/0.1% Tween-20. The IL-1 *β* ELISA described above was performed to determine which slices contained matured/cleaved IL-1*β*. Additionally, slices underwent western blotting. Slices were placed in the wells of a 13.5% SDS-polyacrylamide gel and underwent electrophoresis, and proteins were then transferred to a PVDL membrane and probed for IL-1*β*. Finally, gel slices were also submitted to the University of Wisconsin Biotechnology Center for mass spectrometry-based proteomics by in-gel digestion followed by nanoLC-MS/MS to identify potential proteinases for IL-1*β*. Protein annotations, identification of significance, and spectral-based quantification were done with the help of Scaffold software (version 4.3.2, Proteome Software Inc., Portland, OR, USA). Protein identifications were accepted if they could be established at greater than 80.0% probability within a 1% false discovery rate and contained at least 2 identified peptides. Protein probabilities were assigned by the ProteinProphet algorithm [43]. Proteins that contained similar peptides and could not be differentiated based on MS/MS analysis alone were grouped to satisfy the principles of parsimony.

### 2.12. THP-1 Cell Line

The monocyte cell type, THP-1, was obtained from ATCC (ATCC, Manassas, VA, USA) and cultured in a medium similar to the eosinophil culture medium described above (RPMI and 10% FBS). THP-1 was differentiated into macrophages by overnight incubation with 100 nM PMA and 10 ng/ml of IFN-*γ* (R&D Systems). THP-1 was then pretreated (30 min), similarly as for eosinophils, with pharmacological inhibitors of caspase-1 (Z-WEHD-FMK, 5 *μ*M) or MMP-9 (MMP-9 inhibitor 1, 5 *μ*M), or vehicle alone, before activation with LPS (100 ng/ml; Sigma-Aldrich Corp., St. Louis, MO, USA) for 24-48 h. Cell supernatants were stored at -80°C before analysis of IL-1*β* release by ELISA.

### 2.13. Statistical Analysis

Statistical analysis was performed using SigmaStat software (Systat Software Inc., Chicago, IL, USA). Comparison of different treatments in paired samples was performed using the *t*-test and the Mann–Whitney rank sum test (for not normally distributed values). For comparison of more than 2 groups, one-way analysis of variance followed by the Holm-Sidak method were used. A *p* value of <0.05 was considered significant.

## 3. Results

### 3.1. Activated Eosinophils Express and Stabilize *IL1B* mRNA

**Figure 1:**
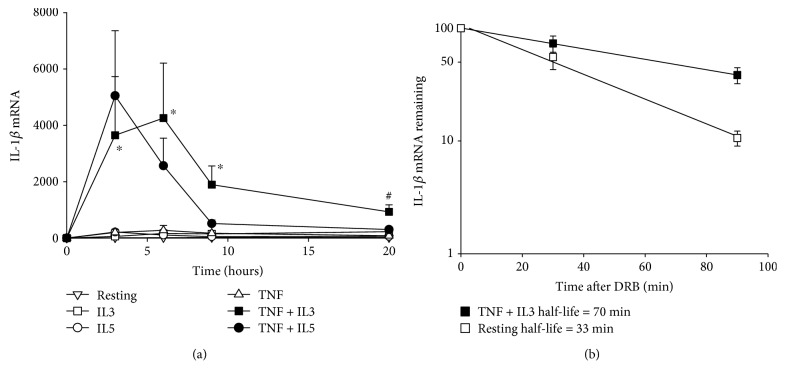
Activated eosinophils express a high level of IL-1*β* mRNA. (a) Blood eosinophils were cultured for 0 (T0), 3, 6, 9, and 20 h with medium, (resting) TNF-*α* (TNF), IL-3 (IL3), IL-5, TNF-*α* plus IL-3 (TNF + IL3), or TNF-*α* plus IL-5 (TNF + IL5). Levels of *IL1B* mRNA were determined by RT-qPCR, normalized to *GUSB* and expressed as fold change (2^−*∆∆*Ct^) from T0. Data are mean ± SEM of experiments on eosinophil preparations from three subjects. ^∗^ *p* < 0.05 for TNF-*α* plus IL-3 versus resting, IL-3, IL-5, or TNF-*α* alone or IL-5 plus TNF-*α*; ^#^ *p* < 0.05 for TNF-*α* plus IL-3 versus resting, IL-5, and TNF-*α*, at corresponding time points. (b) Eosinophils were cultured with medium alone (resting) or TNF-*α* plus IL-3 for 4.5 h, before the addition of DRB. After DRB addition, cells were harvested at T0, 30, and 90 min and *IL1B* mRNA was quantified by RT-qPCR. Data were normalized to *GUSB* and expressed as the percentage of mRNA remaining compared to T0. Data are presented as the mean of experiments on eosinophil preparations from 4 donors. The half-life time of *IL1B* mRNA for both conditions is indicated in the legend on the graph.

Circulating blood eosinophils activated with TNF-*α* plus a common *β*-chain family cytokine (IL-5 or IL-3) expressed a high level of *IL1B* mRNA (Figure 1(a)). In TNF-*α* plus IL-3-activated cells, the maximum expression of *IL1B* mRNA was maintained from 3 to 6 hours after the beginning of activation, and the level remained high after 9 h.  Figure 1(b) shows that between 3 and 6 hours (4.5 h) after the beginning of activation, *IL1B* mRNA was stabilized in TNF-*α* plus IL-3-activated eosinophils compared to inactivated (resting) cells, with a half-life time of 70 min versus 33 min, respectively.

### 3.2. Activated Eosinophils Release Bioactive IL-1*β* Protein with an Apparent Size < 17 kDa

**Figure 2:**
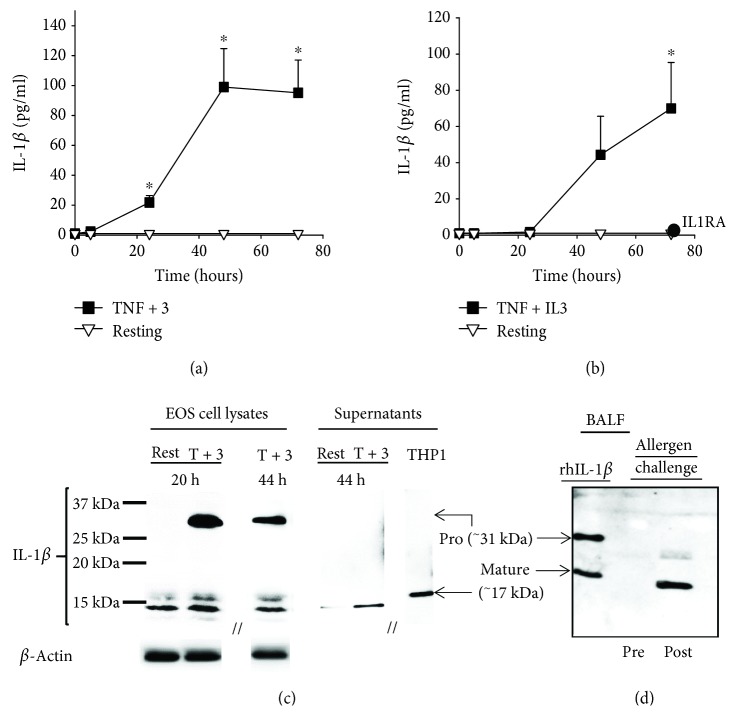
Activated eosinophils release bioactive IL-1*β* protein with a MW < 17 kDa. (a and b) Blood eosinophils were cultured with medium alone (resting) or TNF-*α* plus IL-3 (TNF + 3) for 5, 24, 48, and 72 h. (a) IL-1*β* present in eosinophil cultures was measured by ELISA. (b) The bioactivity of IL-1*β* was determined using HEK-Blue™ IL-1*β* reporter cells and interpolation on a standard curve produced using a recombinant human mature IL-1*β*. The addition of IL1RA (0.4 *μ*g/ml) to the reporter cells completely inhibited the bioactivity of the conditioned media obtained 72 h after eosinophil stimulation with TNF + IL3. Data are mean ± SEM of experiments on eosinophil preparations from five to six subjects (a) and three subjects (b). ^∗^ *p* < 0.05 for TNF-*α* plus IL-3 versus resting, at the corresponding time point. (c) Blood eosinophils were cultured with medium alone (rest) or TNF-*α* plus IL-3 (T + 3) for 20 and 44 h. Cell lysates and concentrated conditioned media (supernatants) were prepared. Western blots were performed to visualize IL-1*β* protein sizes. Supernatant fluid from activated THP-1 were used as a positive control for the 17 kDa mature form of IL-1*β*. The blot shown is representative of three using three different blood eosinophil donors. (d) BAL fluids (BALF) obtained by bronchoscopy before (Pre) and 48 h after (Post) a segmental allergen challenge were evaluated by western blot for the presence and size of IL-1*β*. Positive control (rhIL-1*β*) includes both pro- and mature forms of IL-1*β*. The blot is representative of two experiments using BAL fluids from two different subjects.

Unlike TNF-*α* plus IL-5 (not shown), TNF-*α* plus IL-3-activated blood eosinophils released a mature form of IL-1*β*, measurable by ELISA (Figure 2(a)). Maximum production was observed starting 48 h after the beginning of culture and was maintained for at least 72 h. At 72 h, IL-1*β* bioactivity was also significantly detected using a HEK-Blue™ IL-1*β* reporter cell line (Figure 2(b)). The specificity of the activity was further demonstrated by its inhibition using IL1RA, which competes with IL-1*β* to bind the IL-1 receptor (Figure 2(b)). Figure 2(c) shows that a proform of IL-1*β* was readily visible in eosinophils 20 h and 44 h after the beginning of activation with TNF-*α* plus IL-3. In addition, multiple different sizes of IL-1*β* were also identified, all with an apparent MW < 17 kDa (Figure 2(c)). The strongest band just below 15 kDa was also detected by western blot in supernatants from eosinophils activated for 44 h (Figure 2(c)), while no detection was observed in this condition at 20 h (not shown). Markedly, IL-1*β* with an apparent size of <17 kDa was also detected in BAL fluids 48 h after mild asthmatic subjects had received a segmental allergen challenge (Figure 2(d)).

### 3.3. Identification of Matrix Metalloproteinase-9 (MMP-9) as a Potential Protease to Cleave IL-1*β* in Activated Eosinophils

**Figure 3:**
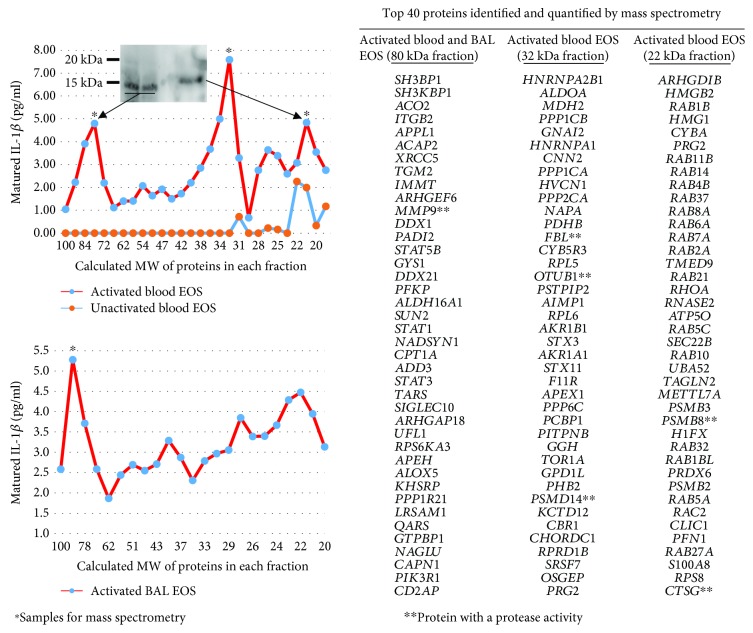
Identification of matrix metalloproteinase-9 (MMP-9) as a potential protease for IL-1*β* cleavage in cytokine-activated eosinophils. Blood and BAL eosinophils were cultured in medium alone (inactivated) or with TNF-*α* plus IL-3 (both at 10 ng/ml; activated) for 48 h and 72 h, respectively. Using a zymography approach, eosinophil lysates were electrophoresed in a 12% SDS-PAGE gel copolymerized with pro-IL-1*β*. The pro-IL-1*β* zymograms were renatured and incubated overnight under conditions compatible with the activation of proteases. Each lane of the pro-IL-1*β* zymogram was excised and sequentially cut into 1 mm slices. The sizes of proteins (MW) in each gel slice are shown on the *x*-axis of the graphs. Proteins were allowed to diffuse out of the gel slices. The *y*-axis of the graphs indicates the amount of matured/cleaved IL-1*β* present in each gel slice as measured by ELISA. As shown on the upper graph, gel slices underwent western blotting for IL-1*β* to identify the size of the IL-1*β* proteins. Western blot using the ~80 kDa gel slice from two experiments using blood eosinophils from two different subjects is shown, and it identifies the IL-1*β* with a size of ~15 kDa. The ~80 kDa, ~32 kDa, and ~22 kDa (^∗^) gel slices were also submitted to the University of Wisconsin Biotechnology Center for mass spectrometry-based proteomics. On the right side of the figure are listed the top 40 genes coding for identified proteins. The lists of genes coding for proteins present in (i) the ~80 kDa gel slice for both activated blood and BAL eosinophils (*n* = 2), (ii) the ~32 kDa gel slice in activated blood eosinophils (*n* = 2), and (iii) the ~22 kDa gel slice in activated eosinophils (*n* = 1) are shown. Genes with asterisk possess a protease activity according to DAVID Bioinformatic Resources 6.8 (beta) (National Institute of Allergy and Infectious Diseases (NIAID), NIH) [65].

**Figure 4:**
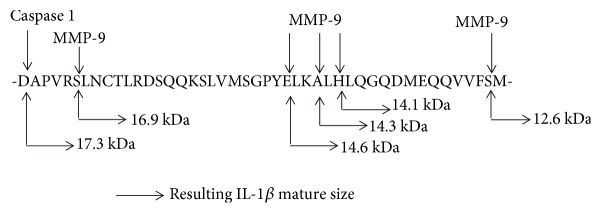
Identification of potential cleavage sites for MMP-9 in IL-1*β*. Potential cleavage sites for MMP-9 resulting in <17 kDa matured IL-1*β* sizes were identified using http://cleavpredict.sanfordburnham.org [41].

Blood and BAL eosinophils were activated with TNF-*α* plus IL-3 for 48 or 72 h, and cell lysates were loaded on a pro-IL-1*β*-containing SDS-PAGE gel (Figure 3). Using this zymography approach, we found 3 gel fractions possessing a protease activity toward pro-IL-1*β*. Proteins in these 3 gel fractions had an apparent MW of ~80 kDa (57 proteins identified by mass spectrometry-based proteomics), ~32 kDa (85 proteins), and ~22 kDa (48 proteins) (Figure 3). The exhaustive lists of proteins found in the ~80 kDa and ~32 kDa fractions are presented in supplemental Tables E1 and E2. The cleavage activity on IL-1*β* in the ~80 kDa gel fraction was found in both blood and BAL eosinophils, and the size of IL-1*β* in this fraction was <17 kDa (~15 kDa) (Figure 3, western blot inset). In both blood and BAL eosinophils, MMP-9, which has a molecular weight of 82 kDa, was listed as highly abundant in the ~80 kDa fraction (Figure 3). As predicted on http://cleavpredict.sanfordburnham.org [44], multiple potential sites in IL-1*β* exist for MMP-9, including 3 sites around ELKA, a well-known cleavage site for MMPs [45], that would generate IL-1*β* sizes between 15 kDa and 14 kDa (Figure 4).

### 3.4. MMP-9 Is Involved in the Release of Mature IL-1*β* by Activated Eosinophils

**Figure 5:**
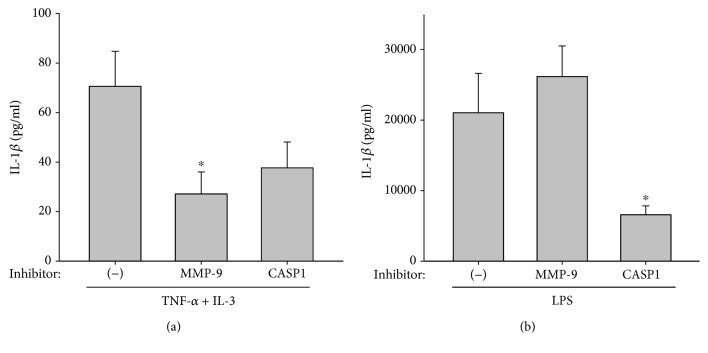
MMP-9 activity is involved in IL-1*β* release by activated eosinophils. (a) Blood eosinophils were cultured with TNF-*α* plus IL-3 (10 ng/ml) for 48 h after a 30 min treatment with Z-WEHD-FMK (caspase-1 (CASP1) inhibitor) or MMP-9 inhibitor 1 (MMP-9 inhibitor) used at 5 *μ*M or vehicle alone (-). The amount of matured IL-1*β* in the supernatant culture was measured by ELISA. ∗ indicates that MMP-9 inhibition significantly reduced IL-1*β* compared to no treatment (-) (*p* < 0.05, *n* = 7 different subjects (Table E3)). (b) The THP-1 cell line was differentiated into macrophages using PMA and IFN-*γ* overnight. Cells were treated with caspase-1 inhibitor (CASP1), MMP-9 inhibitor (MMP9), and vehicle alone (-), the same as for blood eosinophils in (a), and were activated with LPS (100 ng/ml) for 24 h. Matured IL-1*β* released by THP-1 cells was measured by ELISA. ∗ indicates that caspase-1 inhibition significantly reduced IL-1*β* release compared to (-) and MMP-9 (*p* < 0.05, *n* = 5).

Blood eosinophils were prepared from seven subjects (4 females and 3 males, 18 to 53 years old, with allergy). Eosinophils were pretreated with inhibitors of MMP-9 and caspase-1, or vehicle alone, before activation with TNF-*α* plus IL-3. The MMP-9 inhibitor significantly reduced the release of IL-1*β* by activated eosinophils (Figure 5(a)) in all 7 subjects. The caspase-1 inhibitor seemed to also inhibit IL-1*β* release (Figure 5(a)), although it did not reach statistical significance due to variability among eosinophil donors, including eosinophils from 3 subjects who did not (2 subjects) or only slightly (1 subject with <15% inhibition) responded to the caspase-1 inhibitor. This suggests heterogeneity among subjects regarding the role of the inflammasome in the release of IL-1*β* by eosinophils. Conversely, used as a control for the pharmaceutical inhibitors, the monocyte/macrophage cell line THP-1 activated with LPS showed that the release of mature IL-1*β* was inflammasome/caspase-1-dependent as the caspase-1 inhibitor strongly diminished the release of IL-1*β* while the MMP-9 inhibitor was totally ineffective (Figure 5(b)).

## 4. Discussion

Although there are a few reports showing that eosinophils are a source of IL-1*β* [18, 35, 36], no studies have shown the mechanisms of IL-1*β* expression, maturation, and release by eosinophils. By the present study, we demonstrate that cytokine-activated eosinophils stabilize and accumulate *IL1B* mRNA, and then they mature and release IL-1*β* protein in a MMP-9-dependent manner.

In a cell-free system, the matrix metalloproteinases MMP-3 and MMP-9 have been reported as IL-1*β*-converting enzymes, with MMP-9 generating a bioactive IL-1*β* with a MW < 17 kDa [46]. While MMP-3 expression was not detected in activated eosinophils, we have previously reported that TNF-*α* plus IL-3-activated eosinophils produce and release a large amount of pro-MMP-9 protein starting between 24 and 48 h after the beginning of activation, with levels reaching as much as 30 ng/ml after 72 h [37]. This particular time point for MMP-9 production is in agreement with the kinetics of IL-1*β* maturation and release monitored in the present study, where 48 and 72 h of activation were required to detect significant amounts of active IL-1*β* in eosinophil supernatants. However, it is uncertain within these conditions how the pro-MMP-9 form was converted into an active form that was able to cleave IL-1*β*. Eosinophils stimulated to undergo basement membrane transmigration produce the active 84 kDa form of MMP-9 [47–49]. Yet, we did not detect a significant amount of active MMP-9 released from TNF-*α* plus IL-3-activated eosinophils [37]. It is however possible that a portion of MMP-9 matures intracellularly in eosinophils. Notably, the protease, cathepsin G (*CTSG*), was detected by mass spectroscopy in the 22 kDa fraction of our zymogram gel. Cathepsin G is an activator of pro-MMPs, particularly MMP-9 [50], and its production in eosinophils has been previously reported [51, 52].

As mentioned above, it is known that in a cell-free system, MMP-9 generates a bioactive IL-1*β* with an apparent MW < 17 kDa [46]. Analysis using http://cleavpredict.sanfordburnham.org [44] indicates that MMP-9 may in fact cleave IL-1*β* in many different sites, all generating an IL-1*β*fragment < 17 kDa. In the present study, we did indeed observe multiple IL-1*β* apparent sizes < 17 kDa by western blot. Even though the most abundant cleaved form of IL-1*β*, which was present both in cell culture *in vitro* and in BAL fluids after *in vivo* allergen challenge, migrated slightly below 15 kDa, other signals were also detected with apparent sizes around 14 kDa and 16 kDa. Although the ELISA used in this study has little cross-reactivity (<7%) with the proform of IL-1*β*, it is not known how much of each different matured IL-1*β* form that was detected by western blot (between 14 and 16 kDa) was also detected by ELISA with sufficient efficiency. Nevertheless, the ELISA measured as much IL-1*β* as our bioactivity assay performed on the IL-1*β*-responsive cell line, suggesting that the ELISA did not underestimate the amount of bioactive IL-1*β* released from activated eosinophils. It is also interesting to note that the kinetics of IL-1*β* generation as detected by ELISA and the bioactivity assay are not superimposable since the maximum bioactivity was observed 24 h after the maximum IL-1*β* release detected by ELISA. Therefore, the possibility of further maturation of IL-1*β* after being released from eosinophils and the exact IL-1*β* cleavage sites in eosinophils remain unknown.

Besides MMP-9, our zymography analysis demonstrated the presence of an unidentified protease in the 32 kDa fraction of the gel. In addition, detection of a protease activity for IL-1*β* in the 22 kDa fraction of our zymogram gel suggested an inflammasome-dependent and caspase-1 (~20 kDa) activity in both activated and resting eosinophils. While we did not detect caspase-1 protein in this fraction by mass spectrometry, we observed that the inhibition of caspase-1 reduced IL-1*β* release by TNF-*α* plus IL-3-activated eosinophils obtained from most of the subjects included in this study. This indicates that caspase-1 may also have a role in IL-1*β* production in eosinophils; at least in a subpopulation of allergic individuals that remains uncharacterized to that point due to the limited number of subjects included (*n* = 7). The identification and characterization of the subjects whose eosinophils use caspase-1 to mature IL-1*β* are of interest, but it would require a larger number of individuals, which is beyond the scope of the present study. The implication of caspase-1 in certain subjects is however in agreement with previous studies that reported IL-1*β* converting enzyme activity and the presence of the p10 subunit of caspase-1 in resting human eosinophils [53, 54]. In addition, zinc oxide nanoparticles induce human eosinophil production of IL-1*β*, which is reversed by the inhibition of caspase-1 [55], and bone marrow-derived murine eosinophils undergo caspase-1-mediated pyroptosis with the release of IL-1*β* [34]. Besides the implication of caspase-1, inflammasome-independent maturation of IL-1*β* by serine proteases has been described in other cell types (neutrophils and mast cells). Most of these inflammasome-independent proteases cleave IL-1*β* into bioactive forms with molecular weights at or slightly above the caspase generated 17 kDa form [56, 57]. The cytotoxic T lymphocyte granule protein, granzyme A, also produces a biologically active form of IL-1*β*, but with a size slightly below 17 kDa [58]. Interestingly, calpain-1 (*CAPN1*) was also detected by mass spectrometry in our 80 kDa fraction. *CAPN1* is known to cleave IL-1*α* into a 17 kDa active form [59], and yet its role on IL-1*β* in our model was not investigated.

Even a modest increase in mRNA stability has a significant impact on protein production [60]. We show here that *IL1B* mRNA levels in activated eosinophils depend on mRNA stability. This is in agreement with previous works showing the stabilization of other transcripts such as *CSF2* [61] and *INHBA* mRNAs in activated eosinophils particularly in TNF-*α* plus IL-3-activated eosinophils [38]. *IL1B* mRNA has already been shown to be stabilized in other types of activated cells such as fibroblasts and monocytic or macrophage cell lines [62, 63] via AUUUA motifs present in the 3′ untranslated region [64]. The involvement of gene transcription into the accumulation of *IL1B* mRNA was not analyzed in the present study.

## 5. Conclusion

**Figure 6:**
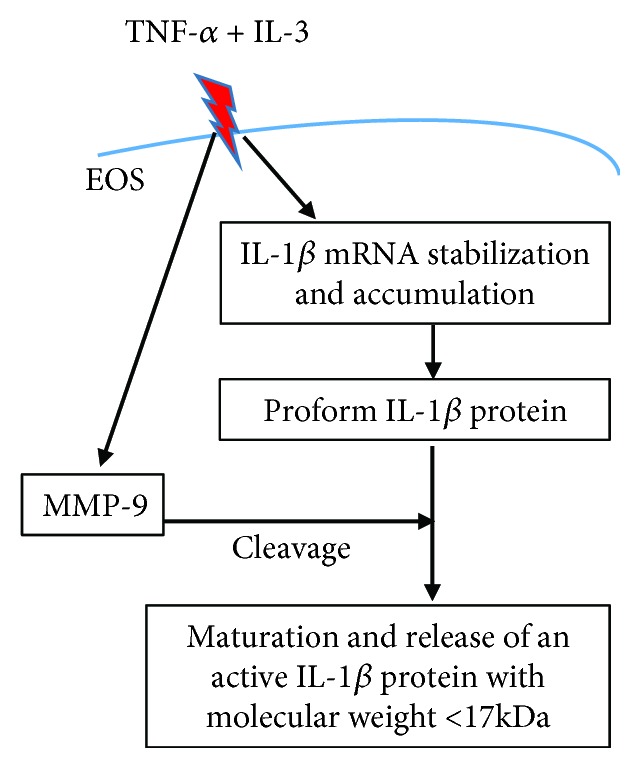
Schematic diagram of the findings. In eosinophils (EOS), TNF-*α* plus IL-3 trigger stabilization and accumulation of IL-1*β* mRNA and IL-1*β* protein production. TNF-*α* plus IL-3 activation also leads to the production of MMP-9, which cleaves the proform of IL-1*β* protein into a mature and active IL-1*β* protein with an apparent molecular weight < 17 kDa.

As summarized in Figure 6, we show here that eosinophils express and produce IL-1*β* with apparent sizes between 16 and 14 kDa. We had previously demonstrated that activated eosinophils produce MMP-9, and we now reveal that MMP-9 produced by activated eosinophils significantly participates in the cleavage and release of a bioactive form of IL-1*β* with an apparent size < 17 kDa. Therefore, via the production of IL-1*β*, eosinophils may participate in profibrotic and proinflammatory events leading, for instance, to a Th17/neutrophilic and treatment-refractory phenotype in asthma.

## Data Availability

The data used to support the findings of this study are available from the corresponding author upon request.

## Abbreviations

BAL: Bronchoalveolar lavage
DRB: 5,6-Dichloro-1-*β*-D-ribofuranosylbenzimidazole
IL-1RA: IL-1*β* receptor antagonist
ILC: Innate lymphoid cells
kDa: kilo Dalton
MMP-9: Matrix metalloproteinase-9
MW: Molecular weight
PMA: Phorbol myristate acetate
Rf: Relative migration distance
Rh: Recombinant human
SBP-Ag: Segmental bronchoprovocation with an allergen
SEM: Standard error of the mean.

## Supplementary Materials

Supplementary Materials
Table E1: proteins detected by mass spectrometry in the ~80 kDa gel fraction that includes an IL-1*β* cleavage activity.  Table E2: proteins detected in the ~32 kDa fraction.  Table E3: characteristics of blood donors used for the preparation of eosinophils included in the analysis shown on Figure 5(a).
