# Supplementary material for: Matrix Metalloproteinase-9-Dependent Release of IL-1β by Human Eosinophils
Source: Mediators Inflamm. 2019 Feb 17;2019:7479107. doi: 10.1155/2019/7479107 (PMC6398033; doi:10.1155/2019/7479107)
Supplement: Supplementary Materials — Table E1: proteins detected by mass spectrometry in the ~80 kDa gel fraction that includes an IL-1β cleavage activity. Table E2: proteins detected in the ~32 kDa fraction. Table E3: characteristics of blood donors used for the preparation of eosinophils included in the analysis shown on Figure 5(a). [file 7479107.f1.docx]

**Supplemental information**

**Matrix Metalloproteinase-9-Dependent Release of IL-1ß by Human Eosinophils**

Esnault, Stephane*, Kelly, Elizabeth A.*, Johnson, Sean H.*, DeLain Larissa P.*, Haedt Madeline J.*, Noll Andrea L.*, Sandbo, Nathan*, Jarjour, Nizar N.*

*University of Wisconsin-Madison School of Medicine and Public Health, Department of Medicine, Division of Allergy, Pulmonary and Critical Care Medicine, Madison, WI, USA.

Address correspondence and reprint requests to Stephane Esnault; Division of Allergy, Pulmonary and Critical Care Medicine, Department of Medicine; 600 Highland Avenue, CSC K4/9; University of Wisconsin, School of Medicine and Public Health; Madison, WI 53792-9988; E-mail: sesnault@wisc.edu; Tel: +1 608- 265-8938; FAX: +1 608-263-3104

**Table E1: Proteins detected by mass spectrometry in the ~80 kDa gel fraction that includes an IL-1ß cleavage activity**

|  |  | **Quantification** | |
| --- | --- | --- | --- |
|  | **MW kDa** | **MS1 Blood** | **MS2 BAL** |
| 3BP1_HUMAN SH3 domain-binding protein 1 GN=SH3BP1 | 76 | 19 | 29 |
| SH3K1_HUMAN SH3 domain-containing kinase-binding protein 1 GN=SH3KBP1 | 73 | 16 | 27 |
| B9A067_HUMAN MICOS complex subunit MIC60 GN=IMMT | 79 | 15 | 15 |
| A2A274_HUMAN Aconitate hydratase, mitochondrial GN=ACO2 | 88 | 13 | 25 |
| XRCC5_HUMAN X-ray repair cross-complementing protein 5 GN=XRCC5 | 83 | 13 | 19 |
| SUN2_HUMAN SUN domain-containing protein 2 GN=SUN2 | 80 | 13 | 9 |
| DP13A_HUMAN DCC-interacting protein 13-alpha GN=APPL1 | 80 | 12 | 21 |
| A0A087X2G1_HUMAN ATP-dependent RNA helicase DDX1 GN=DDX1 | 74 | 11 | 16 |
| KS6A3_HUMAN Ribosomal protein S6 kinase alpha-3 GN=RPS6KA3 | 84 | 11 | 5 |
| ARHG6_HUMAN Rho guanine nucleotide exchange factor 6 GN=ARHGEF6 | 88 | 11 | 19 |
| PADI2_HUMAN Protein-arginine deiminase type-2 GN=PADI2 | 76 | 11 | 16 |
| SYQ_HUMAN Glutamine--tRNA ligase GN=QARS | 88 | 10 | 4 |
| A0A087X1H5_HUMAN Arf-GAP with coiled-coil, ANK repeat and PH domain-containing protein 2 GN=ACAP2 | 88 | 10 | 23 |
| STA5B_HUMAN Signal transducer and activator of transcription 5B GN=STAT5B | 90 | 9 | 17 |
| ITB2_HUMAN Integrin beta-2 GN=ITGB2 | 85 | 8 | 27 |
| STAT1_HUMAN Signal transducer and activator of transcription 1-alpha/beta GN=STAT1 | 87 | 8 | 12 |
| MMP9_HUMAN Matrix metalloproteinase-9 GN=MMP9 | 78 | 8 | 21 |
| GYS1_HUMAN Glycogen [starch] synthase, muscle GN=GYS1 | 84 | 8 | 17 |
| SYTC_HUMAN Threonine--tRNA ligase, cytoplasmic GN=TARS | 83 | 8 | 9 |
| UFL1_HUMAN E3 UFM1-protein ligase 1 GN=UFL1 | 90 | 8 | 8 |
| CD2AP_HUMAN CD2-associated protein GN=CD2AP | 71 | 8 | 4 |
| A16A1_HUMAN Aldehyde dehydrogenase family 16 member A1 GN=ALDH16A1 | 85 | 8 | 15 |
| DDX21_HUMAN Nucleolar RNA helicase 2 GN=DDX21 | 87 | 8 | 16 |
| FUBP2_HUMAN Far upstream element-binding protein 2 GN=KHSRP | 73 | 8 | 8 |
| P85A_HUMAN Phosphatidylinositol 3-kinase regulatory subunit alpha GN=PIK3R1 | 84 | 8 | 5 |
| STAT3_HUMAN Signal transducer and activator of transcription 3 GN=STAT3 | 88 | 8 | 10 |
| PPR21_HUMAN Protein phosphatase 1 regulatory subunit 21 GN=PPP1R21 | 88 | 7 | 8 |
| NADE_HUMAN Glutamine-dependent NAD(+) synthetase GN=NADSYN1 | 79 | 7 | 13 |
| PFKAP_HUMAN ATP-dependent 6-phosphofructokinase, platelet type GN=PFKP | 86 | 7 | 17 |
| C9JIF9_HUMAN Acylamino-acid-releasing enzyme GN=APEH | 82 | 7 | 9 |
| DHB4_HUMAN Peroxisomal multifunctional enzyme type 2 OS=Homo sapiens GN=HSD17B4 | 80 | 6 | 6 |
| LETM1_HUMAN LETM1 and EF-hand domain-containing protein 1, mitochondrial GN=LETM1 | 83 | 6 | 3 |
| TGM2_HUMAN Protein-glutamine gamma-glutamyltransferase 2 GN=TGM2 | 77 | 5 | 25 |
| RHG25_HUMAN Rho GTPase-activating protein 25 GN=ARHGAP25 | 73 | 5 | 3 |
| CAN1_HUMAN Calpain-1 catalytic subunit GN=CAPN1 | 82 | 5 | 8 |
| LOX5_HUMAN Arachidonate 5-lipoxygenase GN=ALOX5 | 78 | 5 | 11 |
| GTPB1_HUMAN GTP-binding protein 1 GN=GTPBP1 | 72 | 5 | 9 |
| RUFY1_HUMAN RUN and FYVE domain-containing protein 1 GN=RUFY1 | 80 | 5 | 3 |
| CUL3_HUMAN Cullin-3 GN=CUL3 | 89 | 4 | 6 |
| LRSM1_HUMAN E3 ubiquitin-protein ligase LRSAM1 GN=LRSAM1 | 84 | 4 | 10 |
| SSRP1_HUMAN FACT complex subunit SSRP1 GN=SSRP1 | 81 | 4 | 8 |
| SIG10_HUMAN Sialic acid-binding Ig-like lectin 10 GN=SIGLEC10 | 77 | 4 | 13 |
| NUP88_HUMAN Nuclear pore complex protein Nup88 GN=NUP88 | 84 | 4 | 6 |
| CPT1A_HUMAN Carnitine O-palmitoyltransferase 1, liver isoform GN=CPT1A | 88 | 4 | 15 |
| CLCN7_HUMAN H(+)/Cl(-) exchange transporter 7 GN=CLCN7 | 89 | 4 | 3 |
| A0A087WX08_HUMAN Gamma-adducin GN=ADD3 | 75 | 4 | 15 |
| HNRPR_HUMAN Heterogeneous nuclear ribonucleoprotein R GN=HNRNPR | 71 | 3 | 6 |
| A0A087WSZ7_HUMAN Dihydropyrimidinase-related protein 3 GN=DPYSL3 | 77 | 3 | 4 |
| B7Z4B8_HUMAN Heterogeneous nuclear ribonucleoprotein U-like protein 1 GN=HNRNPUL1 | 86 | 3 | 8 |
| THMS2_HUMAN Protein THEMIS2 GN=THEMIS2 | 72 | 3 | 6 |
| ARBK1_HUMAN Beta-adrenergic receptor kinase 1 GN=ADRBK1 | 80 | 3 | 2 |
| H3BLZ8_HUMAN Probable ATP-dependent RNA helicase DDX17 GN=DDX17 | 80 | 3 | 2 |
| DC1I2_HUMAN Cytoplasmic dynein 1 intermediate chain 2 GN=DYNC1I2 | 71 | 3 | 2 |
| IKKB_HUMAN Inhibitor of nuclear factor kappa-B kinase subunit beta GN=IKBKB | 87 | 3 | 2 |
| ANAG_HUMAN Alpha-N-acetylglucosaminidase GN=NAGLU | 82 | 2 | 11 |
| E9PKP7_HUMAN Nucleolar transcription factor 1 GN=UBTF | 87 | 2 | 2 |
| RHG18_HUMAN Rho GTPase-activating protein 18 GN=ARHGAP18 | 75 | 2 | 15 |
| CD180_HUMAN CD180 antigen GN=CD180 | 74 | 2 | 4 |

Gel fraction including proteins with a MW ~80 KD and displaying IL-1ß cleavage activity was analyzed by mass spectrometry. MS1 blood was a first mass spectrometry analysis of samples from blood EOS activated for 48 h with TNF plus IL3, while MS2 BAL was a second mass spectrometry of samples from BAL EOS activated for 72 h with TNF plus IL3. The proteins shown in the Table were found in both MS1 and MS2 analysis. Quantification values were normalized to total spectra.

**Table E2: Proteins detected in the ~32 kDa fraction.**

|  |  | **Quantification** | |
| --- | --- | --- | --- |
|  | **MW kDa** | **MS1 Blood** | **MS2 Blood** |
| ROA2_HUMAN Heterogeneous nuclear ribonucleoproteins A2/B1 GN=HNRNPA2B1 | 37 | 26 | 17 |
| ALDOA_HUMAN Fructose-bisphosphate aldolase A GN=ALDOA | 39 | 22 | 17 |
| MDHM_HUMAN Malate dehydrogenase, mitochondrial GN=MDH2 | 36 | 18 | 14 |
| F8W6I7_HUMAN Heterogeneous nuclear ribonucleoprotein A1 GN=HNRNPA1 | 33 | 17 | 7 |
| B4DDF4_HUMAN Calponin-2 GN=CNN2 | 33 | 15 | 9 |
| PP1B_HUMAN Serine/threonine-protein phosphatase PP1-beta catalytic subunit GN=PPP1CB | 37 | 14 | 12 |
| J3KR44_HUMAN Ubiquitin thioesterase OTUB1 GN=OTUB1 | 31 | 13 | 5 |
| PP2AA_HUMAN Serine/threonine-protein phosphatase 2A catalytic subunit alpha isoform GN=PPP2CA | 36 | 13 | 9 |
| PPIP2_HUMAN Proline-serine-threonine phosphatase-interacting protein 2 GN=PSTPIP2 | 39 | 12 | 4 |
| GNAI2_HUMAN Guanine nucleotide-binding protein G(i) subunit alpha-2 GN=GNAI2 | 40 | 12 | 12 |
| HVCN1_HUMAN Voltage-gated hydrogen channel 1 OGN=HVCN1 | 32 | 12 | 10 |
| SNAA_HUMAN Alpha-soluble NSF attachment protein GN=NAPA | 33 | 12 | 10 |
| PP1A_HUMAN Serine/threonine-protein phosphatase PP1-alpha catalytic subunit GN=PPP1CA | 38 | 12 | 11 |
| AIMP1_HUMAN Aminoacyl tRNA synthase complex-interacting multifunctional protein 1 GN=AIMP1 | 34 | 11 | 5 |
| ODPB_HUMAN Pyruvate dehydrogenase E1 component subunit beta, mitochondrial GN=PDHB | 39 | 11 | 9 |
| FBRL_HUMAN rRNA 2'-O-methyltransferase fibrillarin GN=FBL | 34 | 11 | 8 |
| STX11_HUMAN Syntaxin-11 GN=STX11 | 33 | 10 | 3 |
| NB5R3_HUMAN NADH-cytochrome b5 reductase 3 GN=CYB5R3 | 34 | 9 | 9 |
| PIPNB_HUMAN Phosphatidylinositol transfer protein beta isoform GN=PITPNB | 32 | 9 | 2 |
| JAM1_HUMAN Junctional adhesion molecule A GN=F11R | 33 | 9 | 4 |
| RL5_HUMAN 60S ribosomal protein L5 GN=RPL5 | 34 | 8 | 10 |
| ALDR_HUMAN Aldose reductase GN=AKR1B1 | 36 | 8 | 6 |
| TOR1A_HUMAN Torsin-1A GN=TOR1A | 38 | 8 | 3 |
| RL6_HUMAN 60S ribosomal protein L6 GN=RPL6 | 33 | 7 | 7 |
| AK1A1_HUMAN Alcohol dehydrogenase [NADP(+)] GN=AKR1A1 | 37 | 7 | 6 |
| GGH_HUMAN Gamma-glutamyl hydrolase GN=GGH | 36 | 7 | 4 |
| C9JAB2_HUMAN Serine/arginine-rich-splicing factor 7 GN=SRSF7 | 27 | 7 | 2 |
| F8W9Y0_HUMAN Syntaxin-3 GN=STX3 | 30 | 7 | 7 |
| APEX1_HUMAN DNA-(apurinic or apyrimidinic site) lyase GN=APEX1 | 36 | 7 | 6 |
| OSGEP_HUMAN Probable tRNA N6-adenosine threonylcarbamoyltransferase GN=OSGEP | 36 | 7 | 2 |
| MARE2_HUMAN Microtubule-associated protein RP/EB family member 2 GN=MAPRE2 | 37 | 6 | 2 |
| PSDE_HUMAN 26S proteasome non-ATPase regulatory subunit 14 GN=PSMD14 | 35 | 6 | 4 |
| Q5LJA5_HUMAN Ubiquitin carboxyl-terminal hydrolase GN=UCHL5 | 40 | 6 | 2 |
| GPD1L_HUMAN Glycerol-3-phosphate dehydrogenase 1-like protein GN=GPD1L | 38 | 6 | 5 |
| CHRD1_HUMAN Cysteine and histidine-rich domain-containing protein 1 GN=CHORDC1 | 37 | 6 | 3 |
| THTM_HUMAN 3-mercaptopyruvate sulfurtransferase GN=MPST | 33 | 5 | 3 |
| PGP_HUMAN Phosphoglycolate phosphatase GN=PGP | 34 | 5 | 3 |
| PPP6_HUMAN Serine/threonine-protein phosphatase 6 catalytic subunit GN=PPP6C | 35 | 5 | 7 |
| DRA_HUMAN HLA class II histocompatibility antigen, DR alpha chain GN=HLA-DRA | 29 | 5 | 3 |
| CBR1_HUMAN Carbonyl reductase [NADPH] 1 GN=CBR1 | 30 | 5 | 4 |
| PRPS1_HUMAN Ribose-phosphate pyrophosphokinase 1 GN=PRPS1 | 35 | 5 | 2 |
| STX12_HUMAN Syntaxin-12 GN=STX12 | 32 | 5 | 3 |
| PRG2_HUMAN Bone marrow proteoglycan GN=PRG2 | 25 | 5 | 4 |
| PCNA_HUMAN Proliferating cell nuclear antigen GN=PCNA | 29 | 5 | 3 |
| ARK72_HUMAN Aflatoxin B1 aldehyde reductase member 2 GN=AKR7A2 | 40 | 5 | 3 |
| CLIC1_HUMAN Chloride intracellular channel protein 1 GN=CLIC1 | 27 | 5 | 2 |
| TXNL1_HUMAN Thioredoxin-like protein 1 GN=TXNL1 | 32 | 5 | 3 |
| PCBP1_HUMAN Poly(rC)-binding protein 1 GN=PCBP1 | 37 | 5 | 7 |
| KCD12_HUMAN BTB/POZ domain-containing protein KCTD12 GN=KCTD12 | 36 | 4 | 6 |
| FA49B_HUMAN Protein FAM49B GN=FAM49B | 37 | 4 | 2 |
| IPYR_HUMAN Inorganic pyrophosphatase GN=PPA1 | 33 | 4 | 2 |
| RPR1B_HUMAN Regulation of nuclear pre-mRNA domain-containing protein 1B GN=RPRD1B | 37 | 4 | 5 |
| AN32E_HUMAN Acidic leucine-rich nuclear phosphoprotein 32 family member E GN=ANP32E | 31 | 4 | 4 |
| A6NFX8_HUMAN ADP-sugar pyrophosphatase GN=NUDT5 | 26 | 4 | 4 |
| MLEC_HUMAN Malectin GN=MLEC | 32 | 3 | 3 |
| DHRS7_HUMAN Dehydrogenase/reductase SDR family member 7 GN=DHRS7 | 38 | 3 | 4 |
| IPYR2_HUMAN Inorganic pyrophosphatase 2, mitochondrial GN=PPA2 | 38 | 3 | 4 |
| 1B58_HUMAN HLA class I histocompatibility antigen, B-58 alpha chain GN=HLA-B | 40 | 3 | 3 |
| F5GY37_HUMAN Prohibitin-2 GN=PHB2 | 30 | 3 | 7 |
| STX4_HUMAN Syntaxin-4 GN=STX4 | 34 | 3 | 3 |
| TOR1B_HUMAN Torsin-1B GN=TOR1B | 38 | 3 | 2 |
| SCAM3_HUMAN Secretory carrier-associated membrane protein 3 GN=SCAMP3 | 38 | 3 | 4 |
| TDIF1_HUMAN Deoxynucleotidyltransferase terminal-interacting protein 1 GN=DNTTIP1 | 37 | 3 | 4 |
| A6PVN5_HUMAN Serine/threonine-protein phosphatase 2A activator GN=PPP2R4 | 37 | 3 | 3 |
| E7EM64_HUMAN COP9 signalosome complex subunit 6 GN=COPS6 | 36 | 3 | 5 |
| GALE_HUMAN UDP-glucose 4-epimerase GN=GALE | 38 | 3 | 2 |
| PRPS2_HUMAN Ribose-phosphate pyrophosphokinase 2 GN=PRPS2 | 35 | 3 | 3 |
| MYD88_HUMAN Myeloid differentiation primary response protein MyD88 GN=MYD88 | 33 | 3 | 2 |
| H1X_HUMAN Histone H1x GN=H1FX | 22 | 3 | 2 |
| MGLL_HUMAN Monoglyceride lipase GN=MGLL | 33 | 3 | 5 |
| PDXK_HUMAN Pyridoxal kinase GN=PDXK | 35 | 3 | 2 |
| LIMS1_HUMAN LIM and senescent cell antigen-like-containing domain protein 1 GN=LIMS1 | 37 | 3 | 2 |
| ROA0_HUMAN Heterogeneous nuclear ribonucleoprotein A0 GN=HNRNPA0 | 31 | 3 | 3 |
| TM55B_HUMAN Type 1 phosphatidylinositol 4,5-bisphosphate 4-phosphatase GN=TMEM55B | 29 | 3 | 3 |
| ALG5_HUMAN Dolichyl-phosphate beta-glucosyltransferase GN=ALG5 | 37 | 3 | 2 |
| SEC13_HUMAN Protein SEC13 homolog GN=SEC13 | 36 | 3 | 3 |
| SFXN3_HUMAN Sideroflexin-3 GN=SFXN3 | 36 | 3 | 2 |
| SUCA_HUMAN Succinyl-CoA ligase [ADP/GDP-forming] subunit alpha, mitochondrial GN=SUCLG1 | 36 | 2 | 3 |
| IF2A_HUMAN Eukaryotic translation initiation factor 2 subunit 1 GN=EIF2S1 | 36 | 2 | 2 |
| VAPA_HUMAN Vesicle-associated membrane protein-associated protein A GN=VAPA | 28 | 2 | 2 |
| PDCL3_HUMAN Phosducin-like protein 3 GN=PDCL3 | 28 | 2 | 2 |
| BRCC3_HUMAN Lys-63-specific deubiquitinase BRCC36 GN=BRCC3 | 36 | 2 | 3 |
| EMD_HUMAN Emerin GN=EMD | 29 | 2 | 4 |
| HTAI2_HUMAN Oxidoreductase HTATIP2 GN=HTATIP2 | 27 | 2 | 2 |
| AT1B3_HUMAN Sodium/potassium-transporting ATPase subunit beta-3 GN=ATP1B3 | 32 | 2 | 2 |
| C9J7K9_HUMAN Phospholipid scramblase 1 GN=PLSCR1 | 34 | 2 | 2 |

Gel fraction including proteins with a MW ~32 KD and displaying IL-1ß cleavage activity was analyzed by mass spectrometry. MS1 blood was a first mass spectrometry analysis using TNF plus IL3-activated blood EOS for 48 h, while MS2 BAL was a second mass spectrometry using TNF plus IL3-activated BAL EOS for 72 h. The proteins shown in the Table were found in both MS1 and MS2 analysis. Quantification values were normalized to total spectra.

**Table E3: Characteristics of blood donors used for the preparation of eosinophils included in the analysis shown on Figure 5A.**

| **Donor #** | **Age** | **Sex** | **Allergy** | **Asthma** |
| --- | --- | --- | --- | --- |
| **1** | 53 | female | yes | yes |
| **2** | 38 | male | yes | yes |
| **3** | 42 | male | yes | yes |
| **4** | 18 | male | yes | yes |
| **5** | 50 | female | yes | yes |
| **6** | 26 | female | yes | yes |
| **7** | 47 | female | yes | no |
